# Precise Species Detection in Traditional Herbal Patent Medicine, Qingguo Wan, Using Shotgun Metabarcoding

**DOI:** 10.3389/fphar.2021.607210

**Published:** 2021-04-28

**Authors:** Jinxin Liu, Mengmeng Shi, Qing Zhao, Weijun Kong, Weishan Mu, Hongbo Xie, Zhongsi Li, Baoli Li, Linchun Shi

**Affiliations:** ^1^ Hebei Key Laboratory of Study and Exploitation of Chinese Medicine, Chengde Medical University, Chengde, China; ^2^ Institute of Medicinal Plant Development, Chinese Academy of Medical Sciences, Peking Union Medical College, Beijing, China

**Keywords:** shotgun metabarcoding, Qingguo Wan, identification, pharyngitis, traditional patent medicine

## Abstract

As one of the high-incidence diseases in the world, pharyngitis seriously affects the lives of those with the condition. Qingguo Wan is a herbal medicine used for treating pharyngitis, and its quality evaluation is currently only accomplished via traditional identification. However, precise identification becomes challenging with fake products on the market or fungal contamination during the production process. This study used the Illumina NovaSeq platform for targeting the ITS2, *psbA-trnH*, *matK*, and *rbcL* sequences to survey the species composition of lab-made and commercial samples. The results showed that a total of 34.56 Gb of raw data that was obtained represented more than 0.23 billion reads. After assembly, annotation, and operational taxonomic unit clustering, 103, 12, 10, and 12 OTUs were obtained, which belonged to the ITS2, *psbA-trnH*, *matK*, and *rbcL* sequences of the mock lab-made and commercial samples. The analytical results indicated that the sequences of all the prescription ingredients were successfully obtained in the two lab-made samples. The positive control medicinal *Panax quinquefolius* L. sequence was obtained in HSZY175, while *Scutellaria baicalensis* Georgi, *Lonicera japonica* Thunb. *Menispermum dauricum* DC. and *Paeonia lactiflora* Pall. were detected in the three commercial samples. The detection results of the other four herbs in different fragments were not all the same. In addition, a total of 28 fungi OTUs, representing 19 families and 20 genera, were obtained from both the commercial and mock lab-made samples. *Aspergillus, Cladosporium,* and *Penicillium* dominated among the 20 genera. This study demonstrated that the shotgun metabarcoding method is a powerful tool for the molecular identification of the biological ingredients in Qingguo Wan. It can be used to effectively supplement traditional methods while providing a new technique for the quality evaluation of Qingguo Wan.

## Introduction

Pharyngitis is caused by the direct infection of the pharynx and is one of the most common diseases in the world. Continuous industrial development has severely affected the air quality, climate, and environment in recent years. Frequent hazy weather conditions result in an increase in the incidence of pharyngitis and the number of patients requiring treatment. In 2012, Zhang et al. (Hong et al., 2012) reported that about one-third of patients who routinely suffer from pharyngeal discomfort and visit otolaryngology clinics, were displaying chronic pharyngitis. An American medical survey has shown that the number of pharyngitis cases has reached tens of millions every year (Kalra et al., 2016). The inhalation of too much dust and harmful gases, or the frequent consumption of irritating food increases the probability of pharyngitis (Pearce et al., 2020). If the condition is serious, it affects not only the normal functioning of the respiratory system but may also impact the patient’s quality of life. Therefore, it is essential to prevent potentially life-threatening complications. Qingguo Wan is a water honey pill commonly used as a traditional medicine preparation for the treatment of pharyngitis. Qinguo Wan is made from eight kinds of medicinal materials, namely Canarii Fructus (Qingguo), Lonicerae Japonicae Flos (Jinyinhua), Scutellariae Radix (Huangqin), Menispermi Rhizoma (Beidougen), Ophiopogonis Radix (Maidong), Scrophulariae Radix (Xuanshen), Paeoniae Radix Alba (Baishao), and Platycodonis Radix (Jiegeng). It eliminates pharyngeal pain, reducing swelling and relieving pain (Commission, 2020). Several studies have shown that the chemical ingredients in Qingguo Wan prescriptions exhibit excellent anti-inflammatory effects (Shen et al., 2003; Geng et al., 2011; Zhang, 2012; Jia et al., 2016; Nikzad-Langerodi et al., 2017). Of these, Canarii Fructus represents the primary curative ingredient in relieving throat pain, while displaying various pharmacological activities that include antibacterial and anti-inflammatory properties. Furthermore, it has an excellent therapeutic effect on acute pharyngitis (Geng et al., 2011; Jia et al., 2016). Baicalin is a compound obtained from Scutellariae Radix, denoting the main anti-inflammatory ingredient (Shen et al., 2003; Zhang, 2012). Furthermore, the Attenuated Total Reflectance Fourier Transform Infrared (ATR-FTIR) evaluation of the Lonicerae Japonicae Flos extract also showed comparatively good anti-inflammatory activity (Nikzad-Langerodi et al., 2017). The three medicinal herbs mentioned above are used for treating pharyngitis to achieve an optimal therapeutic effect. The remaining five medicinal herbs can increase the efficacy according to the monarch and the minister of traditional Chinese medicine.

Traditional methods, including microscopic identification, thin layer chromatography (TLC) identification, and high-performance liquid chromatography (HPLC) identification have been adopted to detect the seven medicinal ingredients in Qingguo Wan according to the Chinese Pharmacopoeia (Commission, 2020), while the method for identifying Platycodonis Radix has not been described. The microscopic identification method was applied to identify Canarii Fructus, Lonicerae Japonicae Flos, Scutellariae Radix, Scrophulariae Radix, and Paeoniae Radix Alba. TLC was used to identify chlorogenic acid (CGA), paeoniflorin, baicalin, Menispermi Rhizoma, Ophiopogonis Radix, and Scrophulariae Radix. Moreover, the baicalin (Yicun, 2014; Commission, 2020), paeoniflorin (Lei and Futang, 2004), gallic acid (Huowang et al., 2016), and CGA (Yuexin, 2013) content were determined using HPLC technology. Although the above mentioned methods can provide valuable information for the quality control of Qingguo Wan, it is difficult to identify these ingredients through a universal approach and many taxa are difficult to be discriminated among closely related species even if their chemical content tests were qualified (Zhang et al., 2020). Furthermore, most of the traditional methods are depending on human expertize, time-consuming, and they are not appropriate for large-scale computer processing due to the lack of a standardized database (Chen et al., 2014). To overcome such limitations, several methods depending on DNA markers and high throughput sequencing technology have been proposed for evaluation (Lo and Shaw, 2019). In 2018, Xin et al. used HPLC and TLC to identify the active ingredients in Longdan Xiegan Wan (Xin et al., 2018a), and found that the detection results were meeting the Chinese Pharmacopoeia standards. However, the molecular identification technology detected the substitution of Akebiae Caulis (Mutong), Alismatis Rhizoma (Zexie), and the non-prescribed species *Bupleurum marginatum* Wall. ex DC. in the commercial samples. Furthermore, fungi belonging to *Aspergillus* were also revealed by molecular identification technology. The similar results have been found that even though TLC and HPLC tests verified that the products adhered to the existing quality standards, a study by Yufeng Ningxin indicated that substitution with *Pueraria montana* var. *thomsonii* (Benth.) M.R.Almeida and fungal contamination with *Aspergillus* were detected in three herbal products via further DNA metabarcoding (Zhang et al., 2020). An integrated method including macro- and microscopic, chemical and genetic authentication strategies was used to differentiate *Cyanthillium cinereum* from its adulterant *Emilia sonchifolia* (Thongkhao et al., 2020). In addition, some medicinal materials have similar chemical components, and new adulteration based on this phenomenon was found. Han et al. (Zitong et al., 2017) found that the herbal material, Lonicerae Japonicae Flos, was artificially adulterated by Eucommiae Folium in 7% of tested Chinese patent medicines since both Lonicerae Japonicae Flos and Eucommiae Folium contained CGA. It can be seen from the above research that the quality of traditional medicinal materials has always been an issue of global concern. A comprehensive and systematic method is required to accurately identify the original sources of all medicinal materials to ensure the safety and efficacy of clinical medicines.

With the development of high-throughput sequencing technology, the shotgun sequencing approach is a molecular technology mainly used in microbiology. It can be used to analyze mixed samples by randomly breaking genomic DNA, constructing libraries, and non-target sequencing (Quince et al., 2017). Xie et al. (Xie et al., 2013) used shotgun sequencing to study the microbial community structure in different environments during the fermentation process of Shaoxing rice wine, and showed that the community structure and gene function composition changed significantly at different time points. Recent studies by Yang et al. (Xiang et al., 2016) and Arıkan et al. (Arıkan et al., 2020) successfully used this technique to detect changes in the microbial communities in food or beverages. In addition, shotgun sequencing is also widely used in the study of human microbial communities. A randomized clinical study of diabetic patients showed that dietary fiber can specifically increase the beneficial intestinal flora in the human intestine, improving the clinical symptoms of type 2 diabetes while using this technology (Zhao et al., 2018). A study on the influence of American immigration on human intestinal microbiota found that immigration from non-Western countries to Western countries shifted the individual’s microbiome to a more westernized state by using this technology (Vangay et al., 2018). Oh et al. (Oh et al., 2014) successfully applied this technology to the study of human skin microbiota. These studies have shown the feasibility of shotgun technology in identifying complex mixed samples. Xin et al. (Xin et al., 2018a) first used this technology in 2018 by obtaining a small amount of DNA from a sample and analyzing multiple fragments to identify the species in the traditional patent medicine, Longdan Xiegan Wan. The successful application of this technology provides new methods and avenues for identifying the biological ingredients of traditional patent medicine. In 2021, a new approaches combining DNA barcoding and shotgun sequencing was employed for the species identification of Wuhu San and the results showed that this method was potential to effectively complement traditional identification methods (Liu et al., 2021).

In this study, the biological ingredients of Qingguo Wan are identified using shotgun metabarcoding technology. The feasibility of the method is examined using lab-made samples, while the applicability of the technique used for identifying traditional patent medicines is verified with commercial samples, providing a new method for evaluating the quality of Qingguo Wan.

## Materials and Methods

### Sample Collection

**FIGURE 1:**
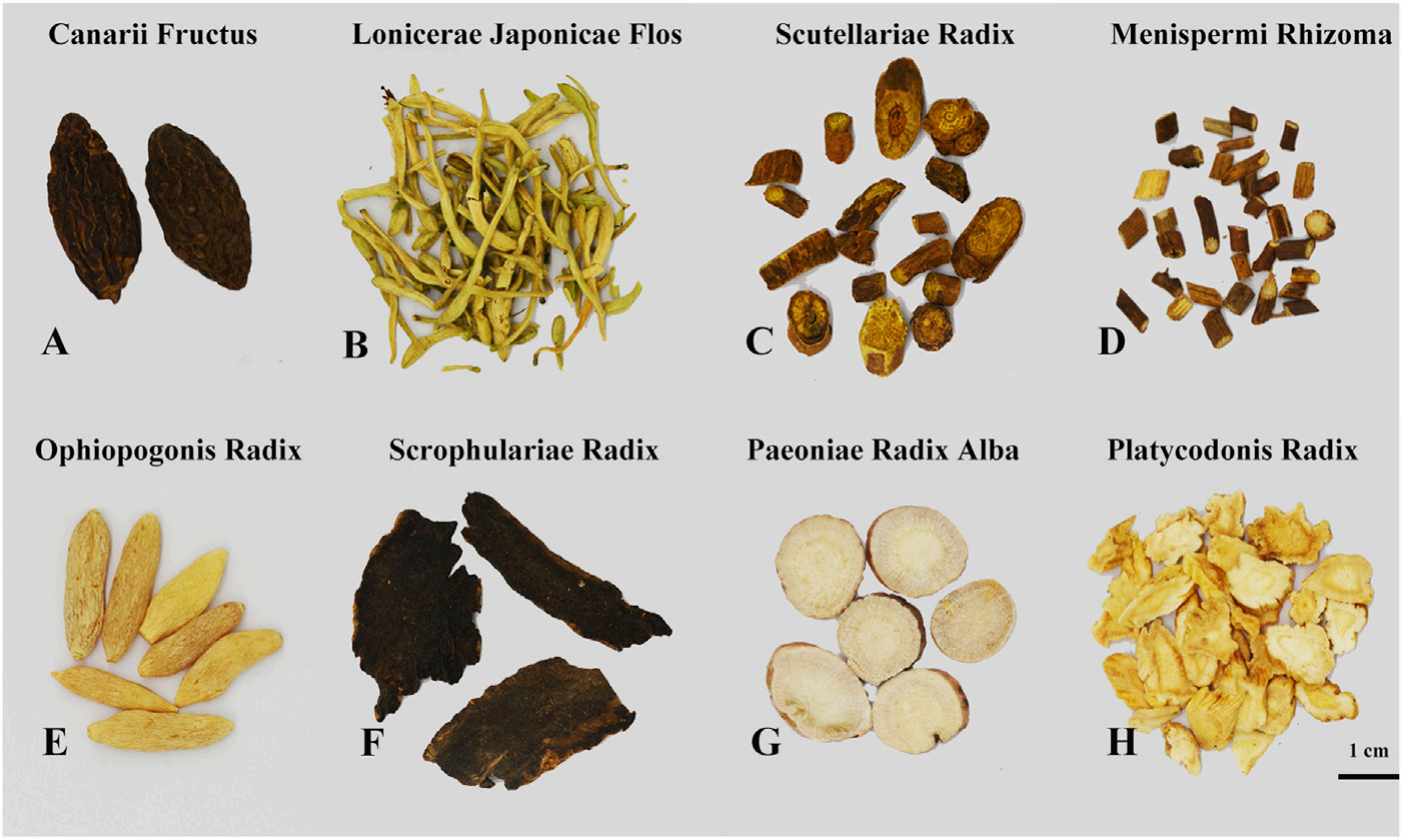
The morphological characteristics of the eight herbal materials found in Qingguo Wan. **(A)** Canarii Fructus (Qingguo), **(B)** Lonicerae Japonicae Flos (Jinyinhua), **(C)** Scutellariae Radix (Huangqin), **(D)** Menispermi Rhizoma (Beidougen), **(E)** Ophiopogonis Radix (Maidong), **(F)** Scrophulariae Radix (Xuanshen), **(G)** Paeoniae Radix Alba (Baishao), and **(H)** Platycodonis Radix (Jiegeng).

Eight medicinal materials including Canarii Fructus (Qingguo, HSYC2064), Lonicerae Japonicae Flos (Jinyinhua, HSYC2058), Scutellariae Radix (Huangqin, HSYC2017), Menispermi Rhizoma (Beidougen, HSYC2053), Ophiopogonis Radix (Maidong, HSYC2047), Scrophulariae Radix (Xuanshen, HSYC2057), Paeoniae Radix Alba (Baishao, HSYC2036), and Platycodonis Radix (Jiegeng, HSYC2048) were collected from Tongrentang Pharmacy and were morphologically authenticated according to the floras of China and the Chinese Pharmacopoeia (Commission, 2020). And they were deposited in the Institute of Medicinal Plant Development herbarium (herbarium code “IMD”, NYBG: https://www.nybg.org/). The eight medicinal materials are shown in Figure 1.

Based on the prescription described in the Chinese Pharmacopoeia, two mock samples of Qingguo Wan were prepared as follows: 1) Eight herbal materials were crushed into powder. 2) The powder was sieved and evenly mixed at the dosage and ratio described in Table 1, and this sample was marked as HSZY163. 3) The Panacis Quinquefolii Radix powder was added to the mixed powder prepared in step 2 as a positive control with the same amount of Qingguo, and this sample was marked as HSZY175. 4) These two powder samples were mixed with honey and water, after which it was molded into pills.

**TABLE 1:** The proportion and dosage of herbal materials listed in the prescription of lab-made and commercial Qingguo Wan according to the Chinese Pharmacopoeia.

Medicinal herbs	Original species	Dosage(g)	Proportion (%)
Canarii Fructus (Qingguo)	*Canarium album* (Lour.) DC.	100	12.5
Lonicerae Japonicae Flos (Jinyinhua)	*Lonicera japonica* Thunb.	100	12.5
Scutellariae Radix (Huangqin)	*Scutellaria baicalensis* Georgi.	100	12.5
Menispermi Rhizoma (Beidougen)	*Menispermum dauricum* DC.	100	12.5
Ophiopogonis Radix (Maidong)	*Ophiopogon japonicus* (Thunb.) Ker Gawl.	100	12.5
Scrophulariae Radix (Xuanshen)	*Scrophularia ningpoensis* Hemsl.	100	12.5
Paeoniae Radix Alba (Baishao)	*Paeonia lactiflora* Pall.	100	12.5
Platycodonis Radix (Jiegeng)	*Platycodon grandiflorus* (Jacq.) A.DC.	100	12.5

In addition, three commercial Qingguo Wan samples were obtained from Tongrentang herbal store and designated as A19 (Lot no. 16035178), HSZY146 (Lot no. 18499001), and HSZY150 (Lot no. 16035178) to examine the applicability of the method in commercial samples.

### DNA Extraction, High-Throughput Sequencing, and Data Analysis

The meta-genomic DNA of traditional herbal patent medicine was extracted according to the previously published protocols of the CTAB-based method (Chunsong et al., 2015) with some changes in the pretreatment steps described by Xin et al. (Xin et al., 2018a). The meta-genomic DNA was tested for integrity via gel electrophoresis and quantified using the NanoDrop ONE ultra-micro spectrophotometer (Thermo Fisher Scientific Inc. USA). Finally, the meta-genomic DNA was sheared into fragments and sequenced using the Illumina NovaSeq platform.

Total DNA was sequenced using the Illumina NovaSeq platform via the shotgun metabarcoding approach. The sequencing adapter and low-quality reads were filtered using Trimmonmatic v0.38 (Bolger et al., 2014). The paired-end reads belonging to ITS2, *psbA-trnH*, *matK,* and *rbcL* were enriched using the local python scripts described by Shi et al. (Shi et al., 2019). The enriched reads belonging to the above four DNA barcoding regions were assembled using MEGAHIT v1.2.9 (Li et al., 2015) and MetaSPAdes v3.13.2 (Nurk et al., 2017) with a value range of k-mer 31–127. The contigs obtained with the two types of software were merged, and duplicates were removed with cd-hit (Li and Godzik, 2006) at 100% identity. The traditional DNA barcoding region of *psbA-trnH*, *matK,* and *rbcL* was acquired by removing the primer sequences using Cutadapt v2.10 (Martin, 2011). The ITS2 regions were determined using a hidden Markov model (HMM)-based annotation methods (Keller et al., 2009). Chimera detection for annotated contigs was performed using UCHIME v4.2 (Edgar et al., 2011). The sequences belonging to each marker were clustered into OTUs at 99% identity using Usearch v11 (https://www.drive5.com/usearch/), and the representative sequences of each OTU were selected for further analysis. The shotgun paired-end reads were mapped to the OTU representative sequences using bowtie2 v2.4.1 (Langmead and Salzberg, 2012), while the sequencing depth and coverage values were calculated using samtools v1.10 (Heng et al., 2009). Poor quality OTUs were removed when its representative sequences displayed a sequencing depth ≤3 and/or coverage ≤95%. The remaining high-quality OTUs were used for species assignment by searching the barcode of traditional Chinese herbal medicine data system (TCM-BOL) (Shilin et al., 2014), the barcode of life data system (BOLD) (Ratnasingham and Hebert, 2007), and GenBank (Clark et al., 2016) databases using the basic local alignment search tool (BLAST) (Camacho et al., 2009). Finally, MEGAN v6.18.9 (Huson et al., 2007) was used for statistics and the taxonomic visualization of the species composition of the traditional herbal patent medicine.

## Results

### High-Throughput Sequencing and Shotgun Metabarcoding Data Assembly

The total DNA of lab-made and commercial samples were sequenced according to a shotgun metabarcoding strategy, and a total of 34.56 Gb of raw data, including more than 230 million paired-end reads, were obtained, which was an average of 6.91 Gb of raw data for each sample. One of the lab-made samples, HSZY163, displayed the most significant amount of sequencing data at 10.81 Gb, while the commercial sample, HSZY150, exhibited the smallest amount of sequencing data at 3.68 Gb. For the five samples, there were 145,688, 2,025,476, 36,135, and 72,051 paired-end reads that were enriched for ITS2, *psbA-trnH, matK,* and *rbcL*, respectively. HSZY175 had the most abundant ITS2 sequencing reads, while HSZY163 had the most paired-end reads belonging to the three chloroplast markers (Table 2).

**TABLE 2:** A summary of the high-throughput sequencing data and the number of reads enriched for ITS2, *psbA-trnH*, *matK,* and *rbcL*.

Sample id	Bases (Gb)	Total reads	The number of enriched reads for each DNA barcode				
			ITS2	*psbA-trnH*	*matK*	*rbcL*	Total
A19	5.9	19,683,174	26,961	292,081	3,859	7,954	330,855
HSZY146	6.19	20,634,727	26,023	401,180	6,494	11,711	445,408
HSZY150	3.68	12,277,349	18,273	281,522	3,782	8,714	312,291
HSZY163	10.81	36,046,154	29,459	546,745	14,280	26,052	616,536
HSZY175	7.98	26,615,513	44,972	503,948	7,720	17,620	574,260

**FIGURE 2:**
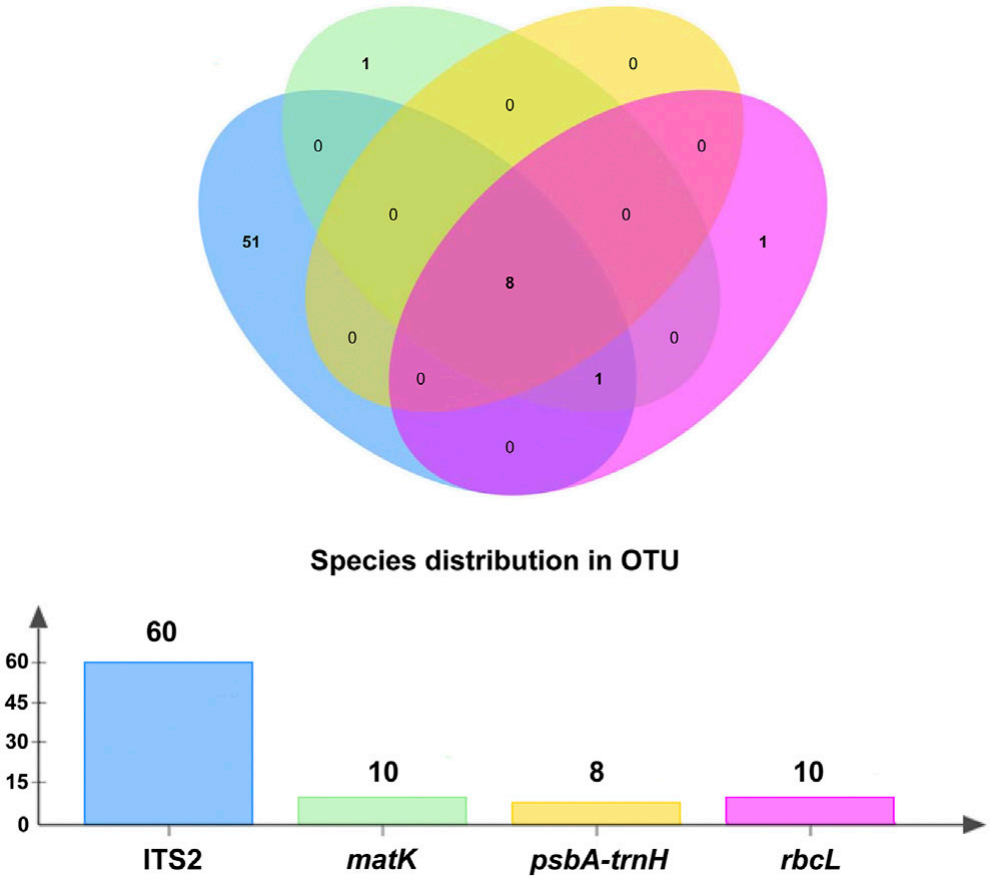
The specific and shared prescribed herbal species identified through ITS2, *psbA-trnH*, *matK,* and *rbcL.*

A total of 4,607 contigs were assembled using MEGAHIT, while 6,450 contigs were assembled using MetaSPAdes. All contigs obtained by the two types of software were combined, and a total of 7,694 unique contigs were acquired after removing redundant sequences. Furthermore, 318 OTUs of traditional ITS2, *psbA-trnH, matK,* and *rbcL* DNA barcoding regions were obtained after removing primers and annotations. A total of 137 OTUs were generated after clustering at a 99% similarity level, of which 103 OTUs belonged to ITS2. The number of OTUs produced by the nuclear ITS2 was approximately three times that of chloroplast *psbA-trnH, matK,* and *rbcL* (Table 3). The number of specific and shared prescription ingredients in Qingguo Wan identified based on ITS2, *psbA-trnH*, *matK,* and *rbcL,* is shown in Figure 2 and Supplementary Tables S1, S2.

**TABLE 3:** The number of contigs of four DNA barcodes in the Qingguo Wan samples.

Parameter	ITS2	*psbA-trnH*	*matK*	*rbcL*
Number of unique contig	417	7102	58	117
Number of DNA barcodes after annotation and chimera detection	196	47	33	42
Number of OTUs	103	12	10	12
Average length (bp)	197.6	477.5	863.1	703
GC%	58.6	29.8	34.5	42.9

### The Species Detection Ability of the Shotgun Metabarcoding for Two Lab-Made Mock Samples

A total of 104 OTUs were obtained for the ITS2, *psbA-trnH*, *matK*, and *rbcL* regions from the two lab-made samples (HSZY163 and HSZY175). For the ITS2 region, there were 20 common OTUs in the two lab-made mock samples, which could be identified to the original species of the eight prescribed herbal ingredients including *Canarium album* (Lour.) DC., *Lonicera japonica* Thunb., *Scutellaria baicalensis* Georgi, *Menispermum dauricum* DC., *Ophiopogon japonicus* (Thunb.) Ker Gawl., *Scrophularia ningpoensis* Hemsl., *Paeonia lactiflora* Pall., and *Platycodon grandiflorus* (Jacq.) A. DC. In addition, it also included some common fungi species, which are described in detail below. For *psbA-trnH*, the same eight OTUs were obtained in both lab-made samples, and their sequences were consistent with the original species of the five ingredients labeled in the prescription. *Lonicera japonica*, *Scrophularia ningpoensis*, and *Paeonia lactiflora* were represented by two OTUs, but that of *Ophiopogon japonicus* was not obtained. Reassessment of the sequence data revealed that the *Ophiopogon japonicus* sequences were present in both samples, but partial sequences were assembled. For *matK*, the same eight OTUs were obtained in both lab-made samples, and their sequences were consistent with the original species of eight ingredients labeled in the prescription. For *rbcL*, the two lab-made samples displayed the same nine OTUs, which all belonged to the original species of the eight prescription ingredients, with *Scrophularia ningpoensis* represented by two OTUs. However, some differences remained between the two lab-made samples, HSZY163 and HSZY175. For example, the *psbA-trnH* OTUs of *Canarium album* and *Scutellaria baicalensis* were present in HSZY163 but could not be obtained in HSZY175. Finally, the ITS2, *psbA-trnH*, *matK*, and *rbcL* for the positive control *Panax quinquefolius* were successfully obtained from HSZY175 (Table 4).

**TABLE 4:** The species detection of the ITS2, *psbA-trnH*, *matK,* and *rbcL* DNA barcoding regions obtained *via* shotgun metabarcoding.

Species	HSZY163				HSZY175			
	ITS2	*psbA-trnH*	*matK*	*rbcL*	ITS2	*psbA-trnH*	*matK*	*rbcL*
*Canarium album*	√	√	√	√	√	—	√	√
*Lonicera japonica*	√	√	√	√	√	√	√	√
*Scutellaria baicalensis*	√	√	√	√	√	—	√	√
*Menispermum dauricum*	√	√	√	√	√	√	√	√
*Ophiopogon japonicus*	√	√	√	√	√	√	√	√
*Scrophularia ningpoensis*	√	√	√	√	√	√	√	√
*Paeonia lactiflora*	√	√	√	√	√	√	√	√
*Platycodon grandiflorus*	√	√	√	√	√	√	√	√
*Panax quinquefolius*	/	/	/	/	√	√	√	√

Note: “√” indicates that the corresponding DNA barcode of this species was obtained, and “—” indicates that the corresponding DNA barcode of this species cannot be obtained, and “/” indicates that this herbal material was not added to the sample.

### The Species Composition of the Commercial Qingguo Wan Samples as Detected by Shotgun Metabarcoding

**FIGURE 3:**
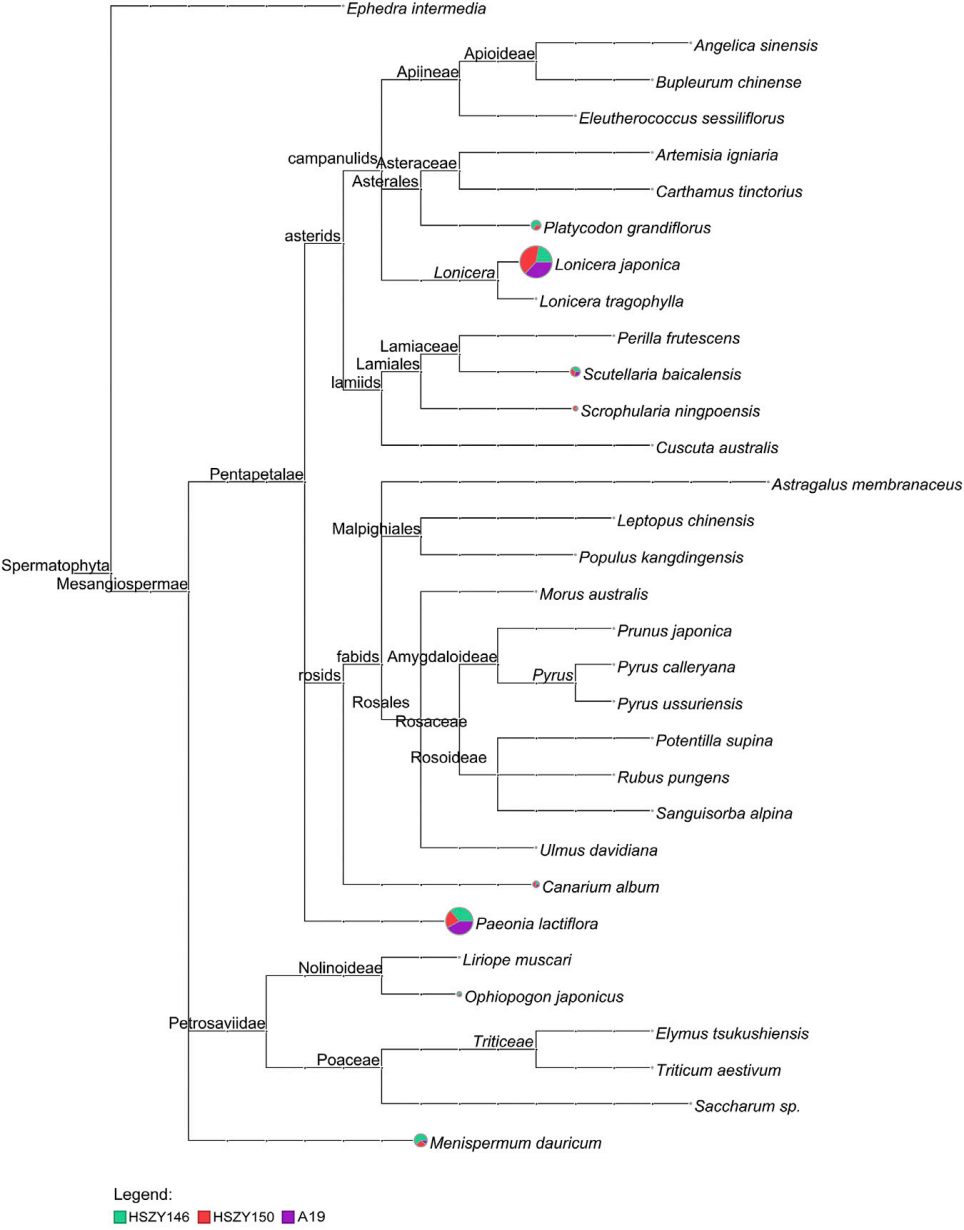
MEGAN6 analysis result for the three samples detected by reads belonging to ITS2 against the NCBI-NT and TCM-BOL database. Each taxonomic node is drawn as a pie chart indicating the proportion of each species in the taxon for each sample.

Three commercial samples were analyzed using the same method as with the lab-made mock samples. Except for *Platycodon grandiflorus*, which was not detected in sample A19, all the original species were detected in all three commercial samples according to the described ingredients for ITS2 and included *Canarium album*, *Lonicera japonica*, *Scutellaria baicalensis*, *Menispermum dauricum*, *Ophiopogon japonicus*, *Scrophularia ningpoensis,* and *Paeonia lactiflora*. Some plant species not listed in the prescription were also detected in the three commercial samples. For example, a total of 140 paired-end reads belonging to *Eleutherococcus sessiliflorus* (Rupr. and Maxim.) S.Y.Hu were detected in A19, accounting for 0.7% of the total paired-end reads of ITS2. One OTU with 71 mapping paired-end reads were detected in sample HSZY146 and was identified as *Elymus tsukushiensis* Honda. A total of 41 *Liriope muscari* (Decne.) L.H.Bailey paired-end reads were detected in the HSZY150 sample, while the sequencing depth was 15.78. The detailed taxonomical content of the three commercial samples detected for ITS2 is shown in Figure 3 and Supplementary Table S3.

**FIGURE 4:**
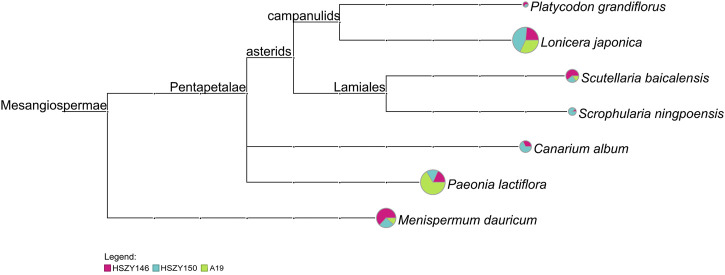
MEGAN6 analysis result for the three samples detected by reads belonging to *psbA-trnH* against the NCBI-NT and TCM-BOL database. Each taxonomic node is drawn as a pie chart indicating the proportion of each species in the taxon for each sample.

When *psbA-trnH* was used for species detection, *Lonicera japonica*, *Scutellaria baicalensis*, *Menispermum dauricum*, and *Paeonia lactiflora* were detected in the three commercial samples. *Canarium album*, *Scrophularia ningpoensis,* and *Platycodon grandiflorus* were detected in HSZY146 and HSZY150. *Ophiopogon japonicus* was not detected in any of the three commercial samples (Figure 4 and Supplementary Table S4).

For *matK* and *rbcL*, *Canarium album*, *Lonicera japonica*, *Scutellaria baicalensis*, *Menispermum dauricum,* and *Paeonia lactiflora* were detected in the three commercial samples. *Platycodon grandiflorus* and Scrophulariaceae were detected in HSZY146 and HSZY150. *Elymus sibiricus* L. was detected in HSZY146, accounting for 1.7% of the total reads of *matK*. *Thinopyrum elongatum* (Host) D.R.Dewey can be detected in HSZY146, accounting for 1.4% of the total *rbcL* reads. *Ophiopogon japonicus* was not detected in A19 and HSZY150. *Scrophularia ningpoensis* and *Platycodon grandiflorus* were not detected in A19 (Supplementary Figures S1, S2 and Supplementary Tables S5, S6).

### The Fungal Contamination of the Lab-Made Mock and Commercial Samples Detected by ITS2

**FIGURE 5:**
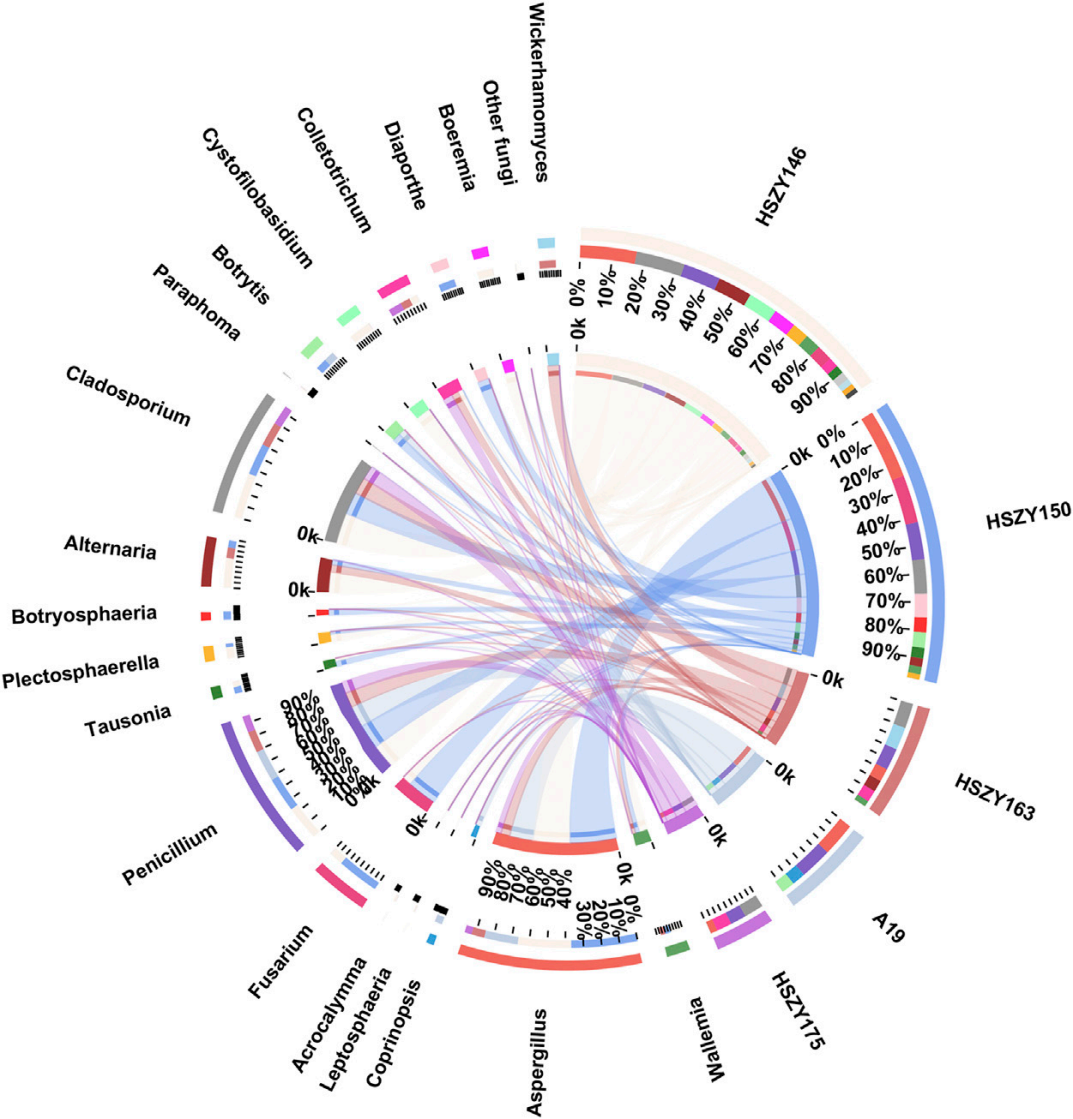
Distribution of the fungi for each sample at the genus level. The data were visualized by Circos. The left half-circle indicates the distribution ratio of species in different samples at the genus level: the outer ribbon represents the species; the inner ribbon represents different groups, and the length represents the sample proportion of a particular genus. The right half-circle indicates the species composition in each sample: the color of the outer ribbon represents samples from different groups; the color of the inner ribbon represents the composition of different species in each sample, and the length of the ribbon represents the relative abundance of the corresponding species (Guo et al., 2020).

A total of 28 fungi OTUs, representing 19 families and 20 genera, were obtained from the two lab-made mock samples and three commercial samples. Taxonomical assignment demonstrated that Aspergillaceae, Cladosporiaceae, and Pleosporaceae represented the most abundant families in the Qingguo Wan samples. Further taxonomical assignment at the genus level indicated that *Aspergillus, Cladosporium,* and *Penicillium* were the predominant of the 20 genera. Fungi belonging to the *Aspergillus* and *Penicillium* genera were detected in all five samples. *Aspergillus* was the most abundant genera in A19, HSZY146, and HSZY150, while *Cladosporium* dominated in HSZY163 and HSZY175 (Figure 5 and Supplementary Table S7).

## Discussion

### The Feasibility of Shotgun Metabarcoding for Detecting Biological Ingredients in Qingguo Wan

In this study, the optimized DNA extraction method can be used successfully to obtain DNA that meets the standard requirements, facilitating effortless shotgun sequencing. The two lab-made and three commercial samples provided the DNA barcode sequences of eight ingredients, obtaining several assembled sequences, which indicated that the shotgun metabarcoding method could be used for species detection in Qingguo Wan. However, there are differences in the identification ability of each marker. In 2010, seven plant DNA barcodes (*psbA-trnH*, *matK*, *rbcL*, *rpoC1*, *ycf5*, ITS2, and ITS) were deeply compared (Chen et al., 2010), the data indicated that ITS2 presented the most suitable region for DNA barcode applications. ITS2, *psbA-trnH,* and *matK* were used to study Longdan Xiegan Wan (Xin et al., 2018a). The results showed that the ITS2 locus exhibited higher identification efficiency, while the *psbA-trnH* region was less effective. In a study to explore DNA barcodes suitable for the identification of Apiaceae, the ITS/ITS2+*psbA-trnH* combination was found to hold considerable potential value (Liu et al., 2014). According to the research mentioned above, ITS2 displayed the strongest distinguishing ability, followed by *psbA-trnH*. And the *rbcL* and *matK* proposed by CBOL Plant Working Group should be adopted for the routine use of DNA barcoding (CBOL Plant Working Group, 2009). A recent study on the identification the TCM preparations, including Bazhen Yimu Wan, Da Huoluo Wan, Niuhuang Jiangya Wan, and Yougui Wan, used ITS2 and *trnL* as targets for herbal materials assessment. The results based on ITS2 showed a higher level of reliability than those of *trnL* at the species level, while the integration of both biomarkers provided higher sensitivity and reliability (Yao et al., 2020). Here, multiple barcode markers were used to improve the results obtained using a single marker for the shotgun metabarcoding analysis. Therefore, this study uses multi-barcode joint identification, which can compensate for the shortcomings of single barcode identification capability, and the results can be verified by different markers.

Some non-prescription ingredients and fungi were found in commercial samples based on ITS2 sequences. The possible reasons for this phenomenon are divided into the following categories: 1) A specific ingredient of another traditional herbal product was accidentally mixed into the Qingguo Wan due to a shared production line. For example, a few sequences belonging to the *Ephedra* genus was detected in the HSZY146 commercial sample. Further analysis showed that the prescription of a herbal product produced by the same company contained medicinal materials derived from the *Ephedra* genus, and accidental cross-contamination was found in a previous report (Xin et al., 2018b). 2) Fungal contamination was introduced during the processes of planting, pre-processing, storage, and transportation. The contamination with fungal species belonging to the genus *Aspergillus*, *Penicillium,* and *Cladosporium* are common in plants, which may produce mycotoxins and have an impact on the efficacy and safety of traditional herbal products (Guo et al., 2020; He et al., 2020; Jiang et al., 2020).

### Difficulties and Challenges Faced by Shotgun Metabarcoding Technology During the Biological Detection of Qingguo Wan

The successful application of shotgun sequencing technology is based on the extraction of high-quality DNA. Traditional medicinal materials contain many ingredients, such as polysaccharides, polyphenols, and various secondary metabolites (Jun et al., 2007; Fanglin and Shulian, 2010; Guanming et al., 2010; Anyu and Wenqin, 2015; Ruilian et al., 2019). In addition, different medicinal parts contain different amounts of DNA, while the types and quantities of chemical substances also vary. These factors affect the quality of the extracted DNA (Kun et al., 2012) and may present significant obstacles when using shotgun sequencing technology to authenticate the ingredients in traditional herbal patent medicine. In this study, high-quality DNA was obtained by optimizing the DNA extraction method of traditional patent medicine, Qingguo Wan (Supplementary Table S8). In addition, there are some variations in the sequencing dataset (e.g. 10.81 Gb for HSZY163 *vs.* 3.68 Gb for HSZY150) due to the problem of uneven mixing in the preparation of the PCR-free library, however, it did not have much impact on the detection results especially for the labeled ingredients as the results of the abundant copies of chloroplast and ITS2 sequences in the plant cells.

Several steps in the bioinformatics pipeline are critical during the process of sequence analysis. For example, correct annotation and primer removal have a significant impact on the results. The *psbA-trnH* sequence coverage of *Scutellaria baicalensis* and *Canarium album* did not reach the 95% threshold in HSZY175. The mapping results indicated that the coverage was unevenly distributed, leading to a certain degree of unreliability in the identification results. As shown in Supplementary Figure S3, the BLAST results of the high coverage region represent the *trnH* conserved sequence, which may be attributed to an error in the annotation or assembly process, failing to remove the *trnH* sequence and producing poor overall mapping results. Therefore, the *Scrophularia ningpoensis* sequence with good mapping results was selected for comparison. As shown in Supplementary Figure S4, the coverage is evenly distributed and continuously extended with high credibility. It is this high sequence coverage that has ensured highly accurate species recovery (Xin et al., 2013). As mentioned above, if errors occur during the annotation process, which is typically caused by a poorly assembled contig, the species identification results will be unreliable. The correct annotation can produce reliable results, as shown in Supplementary Figure S4. Based on this problem, a python script was developed to automate annotation and primer removal, following a manual inspection to ensure the accuracy of the results. It is suggested that future research ensures that the sequence is strictly and accurately annotated and that primers be removed before analysis to eliminate the possibility of false-positive sequences. Analysis problems such as the above mentioned are mainly due to the short-read length generated by second-generation sequencing, which makes sequence assembly and annotation more difficult. The major advantage of third-generation sequencing is the extreme long read length, which can overcome the difficulties of second-generation sequencing. However, the throughput of the third-generation sequencing is low and with high cost. Recently, long-read shotgun metagenomics has been used for studying oral phageome, which revealed that the power in uncovering bacteriophages with enhanced scaffolding, characteristics of their genes, and their interaction with host bacterial immunity (Yahara et al., 2021). It can be seen that long-read metagenomics has broad application prospects if the cost of long-read sequencing can be dropped and sequencing accuracy can be improved.

## Conclusion

This study used shotgun metabarcoding approach to authenticate the biological ingredients of Qingguo Wan, and the results showed that all of the labeled ingredients can be successfully detected by the combination of four frequently used DNA barcodes such as ITS2, *psbA-trnH*, *rbcL* and *matK*. The current study further confirmed that there were differences in the identification efficiency of the four DNA barcodes, and a multi-barcode approach was essential for improving the ability of detecting the species composition in complex herbal products. And, the fungal contamination can be found both in mock and commercial samples with the analysis of ITS2 barcodes obtained from the shotgun sequencing data. Finally, this study firmly showed that shotgun metabarcoding is not only valuable for the quality control of Qingguo Wan, but also can be used for the identification of other traditional herbal products as long as its DNA can be successfully obtained.

## Data Availability

The datasets presented in this study can be found in online repositories. The names of the repository and accession numbers can be found below: NCBI’s Sequence Read Archive (SRA) and SRR12629050, SRR12629049, SRR12629048, SRR12629047, SRR12629046.
