# Supplementary figures and images for: Precise Species Detection in Traditional Herbal Patent Medicine, Qingguo Wan, Using Shotgun Metabarcoding
Source: Front Pharmacol. 2021 Apr 28;12:607210. doi: 10.3389/fphar.2021.607210 (PMC8113863; doi:10.3389/fphar.2021.607210)

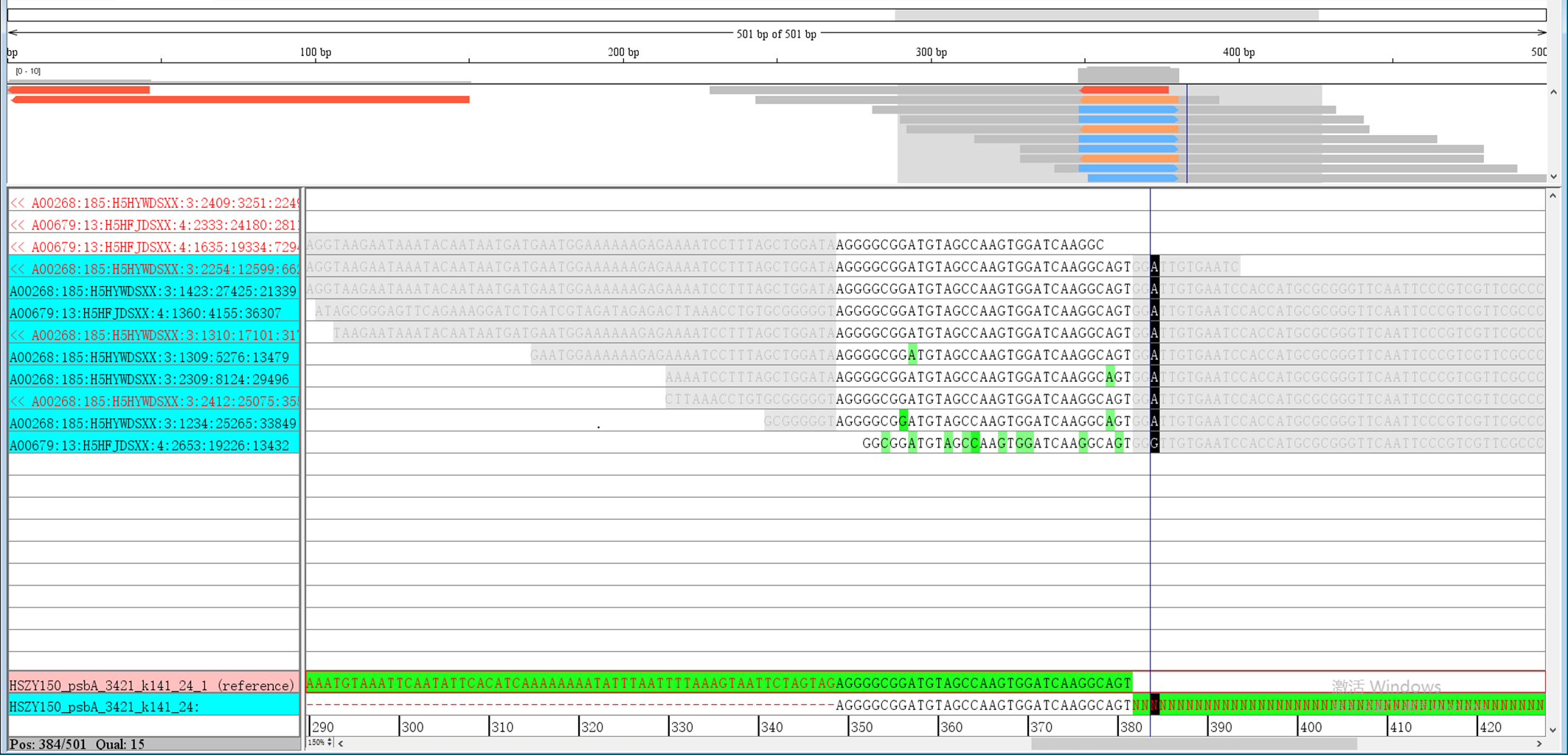

Supplement: Supplementary file 1 [file Image3.TIF]

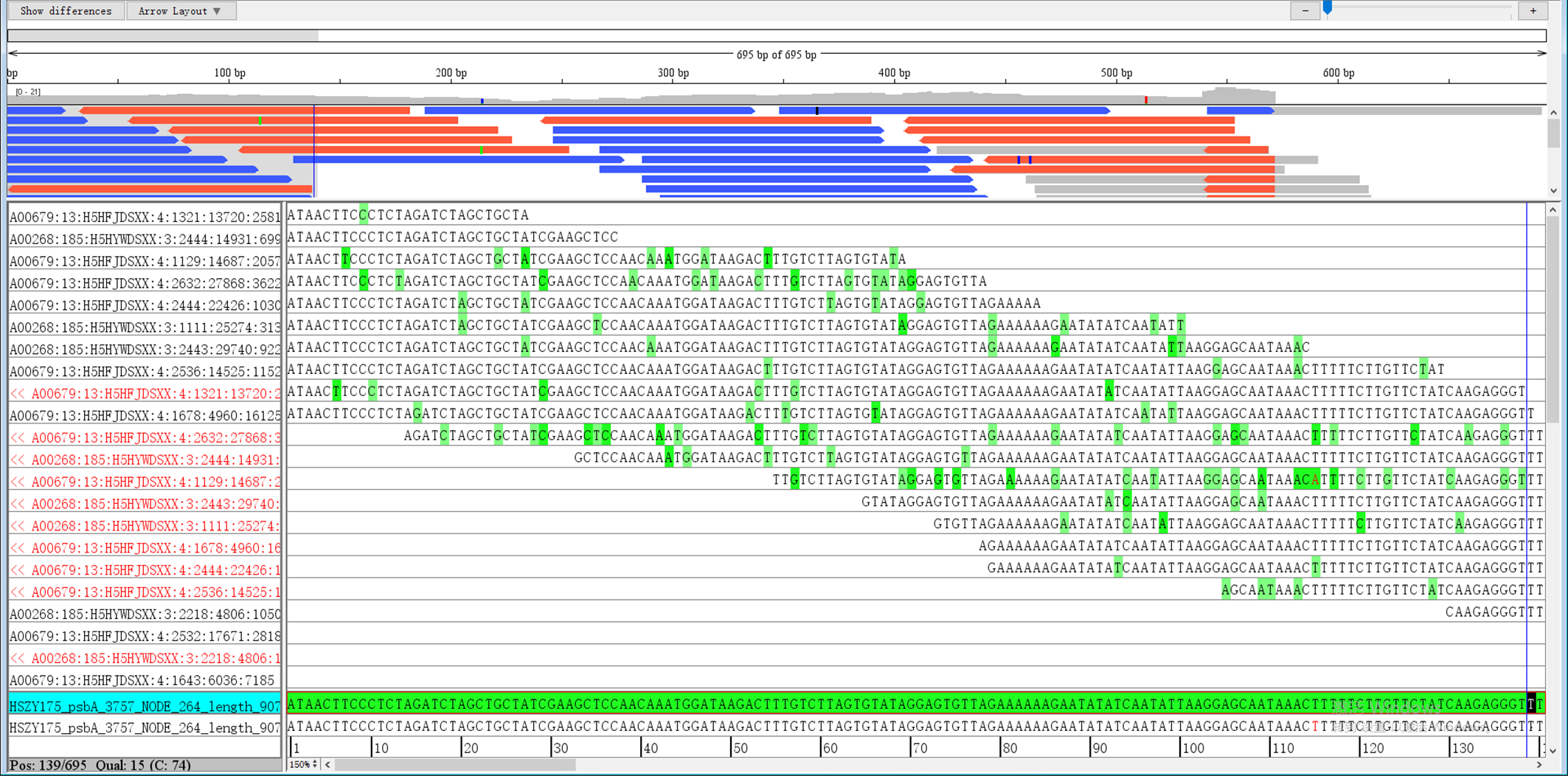

Supplement: Supplementary file 2 [file Image4.TIF]

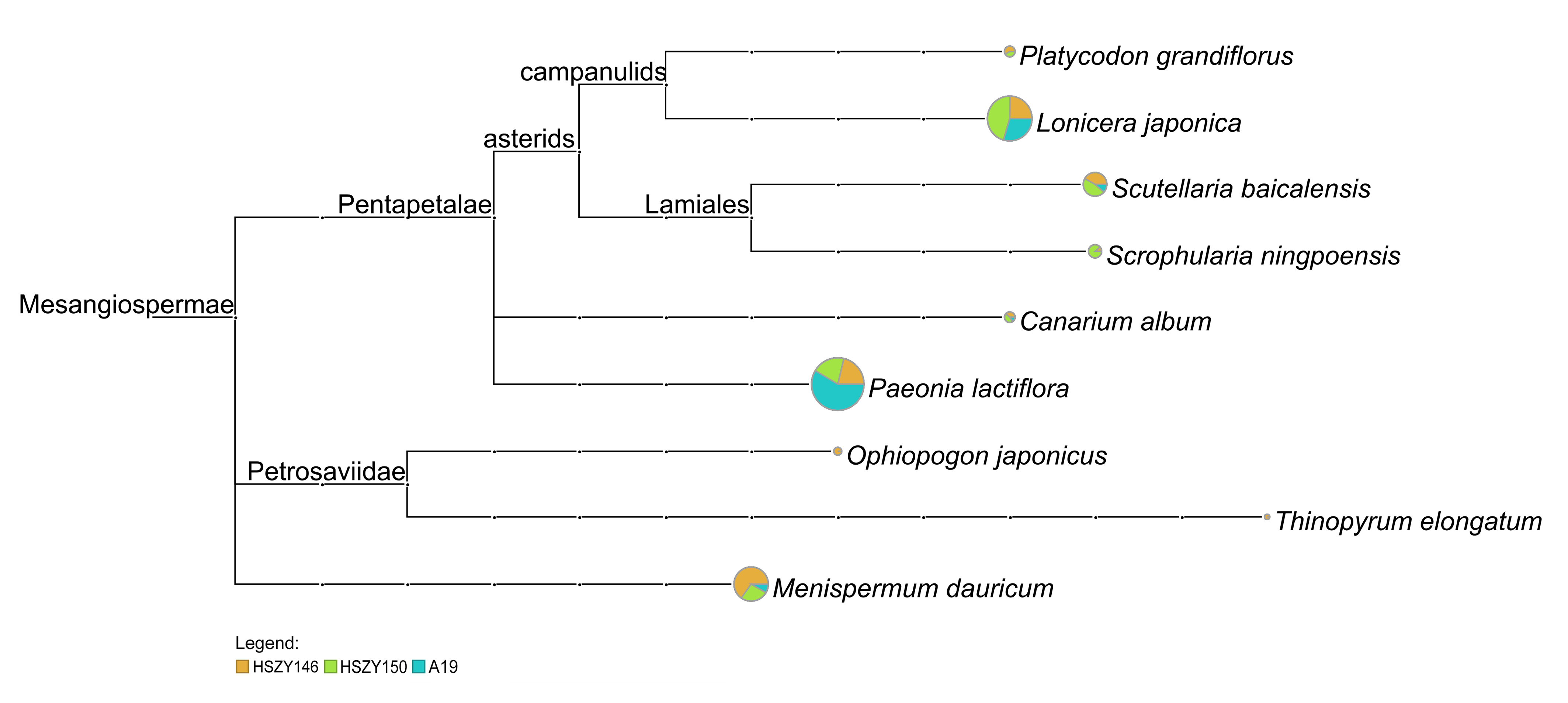

Supplement: Supplementary file 3 [file Image2.TIF]

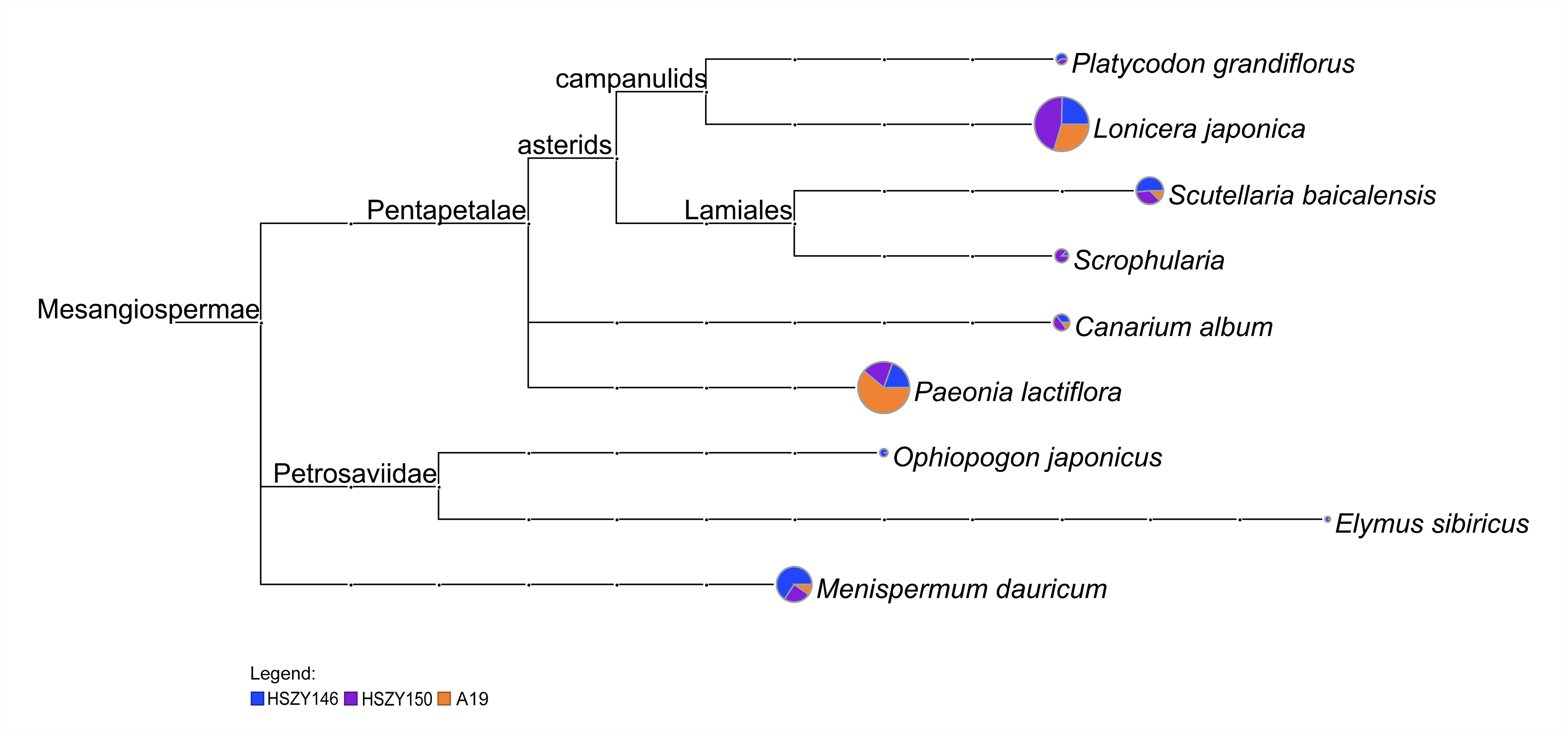

Supplement: Supplementary file 4 [file Image1.TIF]
