# Supplementary material for: Precise Species Detection in Traditional Herbal Patent Medicine, Qingguo Wan, Using Shotgun Metabarcoding
Source: Front Pharmacol. 2021 Apr 28;12:607210. doi: 10.3389/fphar.2021.607210 (PMC8113863; doi:10.3389/fphar.2021.607210)
Supplement: Supplementary file 5 [file DataSheet1.docx]

***Supplementary Material***

## **Supplementary Tables**

**Supplementary Table 1.** OTUs number of species based on ITS2, *psbA-trnH*, *matK* and *rbcL* sequences

| Species | ITS2 | *psbA-trnH* | *matK* | *rbcL* |
| --- | --- | --- | --- | --- |
| *Lonicera japonica* Thunb. | 2 | 2 | 1 | 1 |
| *Paeonia lactiflora* Pall. | 2 | 2 | 1 | 1 |
| *Menispermum dauricum* DC. | 1 | 1 | 1 | 1 |
| *Scutellaria baicalensis* Georgi | 1 | 2 | 1 | 2 |
| *Scrophularia ningpoensis* Hemsl. | 2 | 2 | 1 | 2 |
| *Platycodon grandiflorus* (Jacq.) A.DC. | 1 | 1 | 1 | 1 |
| *Canarium album* (Lour.) DC. | 1 | 1 | 1 | 1 |
| *Ophiopogon japonicus* (Thunb.) Ker Gawl. | 2 | 0 | 1 | 1 |
| *Panax* *quinquefolius* L. | 1 | 1 | 1 | 1 |
| *Liriope muscari* (Decne.) L.H.Bailey | 1 | 0 | 0 | 0 |
| *Elymus tsukushiensis* Honda | 1 | 0 | 0 | 0 |
| *Prunus japonica* Thunb. | 1 | 0 | 0 | 0 |
| *Triticum aestivum* L. | 2 | 0 | 0 | 0 |
| *Potentilla supina* L. | 1 | 0 | 0 | 0 |
| *Populus kangdingensis* Z.Wang & S.L.Tung | 1 | 0 | 0 | 0 |
| *Sanguisorba alpina* Bunge | 1 | 0 | 0 | 0 |
| *Ulmus davidiana* Planch. | 1 | 0 | 0 | 0 |
| *Rubus pungens* Cambess. | 1 | 0 | 0 | 0 |
| *Cuscuta australis* Yunck. | 1 | 0 | 0 | 0 |
| *Pyrus calleryana* Decne. | 1 | 0 | 0 | 0 |
| *Artemisia igniaria* Maxim. | 1 | 0 | 0 | 0 |
| *Pyrus ussuriensis* Maxim. | 1 | 0 | 0 | 0 |
| *Bupleurum chinense* DC. | 1 | 0 | 0 | 0 |
| *Angelica sinensis* (Oliv.) Diels | 1 | 0 | 0 | 0 |
| *Leptopus chinensis* (Bunge) Pojark. | 1 | 0 | 0 | 0 |
| *Lonicera tragophylla* Hemsl. | 1 | 0 | 0 | 0 |
| *Morus australis* L. | 1 | 0 | 0 | 0 |
| *Carthamus tinctorius* L. | 1 | 0 | 0 | 0 |
| *Ephedra intermedia* Schrenk & C.A.Mey. | 1 | 0 | 0 | 0 |
| *Saccharum sp.* | 1 | 0 | 0 | 0 |
| *Astragalus membranaceus* Fisch. ex Bunge | 1 | 0 | 0 | 0 |
| *Perilla frutescens* (L.) Britton | 1 | 0 | 0 | 0 |
| *Eleutherococcus sessiliflorus* (Rupr. & Maxim.) S.Y.Hu | 1 | 0 | 0 | 0 |
| *Acrocalymma sp.* | 1 | 0 | 0 | 0 |
| *Alternaria alternata* | 1 | 0 | 0 | 0 |
| *Aspergillus penicillioides* | 2 | 0 | 0 | 0 |
| *Aspergillus sp.* | 2 | 0 | 0 | 0 |
| *Boeremia exigua* | 1 | 0 | 0 | 0 |
| *Botryosphaeria dothidea* | 1 | 0 | 0 | 0 |
| *Botrytis cinerea* | 1 | 0 | 0 | 0 |
| *Cladosporium sp.* | 1 | 0 | 0 | 0 |
| *Colletotrichum boninense* | 1 | 0 | 0 | 0 |
| *Coprinopsis cinerea* | 1 | 0 | 0 | 0 |
| *Cystofilobasidium capitatum* | 1 | 0 | 0 | 0 |
| *Cystofilobasidium infirmominiatum* | 1 | 0 | 0 | 0 |
| *Cystofilobasidium macerans* | 1 | 0 | 0 | 0 |
| *Diaporthe sp.* | 1 | 0 | 0 | 0 |
| *Dothideomycetes sp.* | 1 | 0 | 0 | 0 |
| *Fusarium fujikuroi* | 1 | 0 | 0 | 0 |
| *Fusarium oxysporum* | 1 | 0 | 0 | 0 |
| *Fusarium solani* | 1 | 0 | 0 | 0 |
| *Fusarium sp.* | 1 | 0 | 0 | 0 |
| *Leptosphaeria sp.* | 1 | 0 | 0 | 0 |
| *Paraphoma sp.* | 1 | 0 | 0 | 0 |
| *Penicillium polonicum* | 1 | 0 | 0 | 0 |
| *Penicillium salmoniflumine* | 1 | 0 | 0 | 0 |
| *Penicillium sp.* | 5 | 0 | 0 | 0 |
| *Plectosphaerella cucumerina* | 1 | 0 | 0 | 0 |
| *Tausonia pullulans* | 1 | 0 | 0 | 0 |
| *Wallemia mellicola* | 1 | 0 | 0 | 0 |
| *Wickerhamomyces sp.* | 1 | 0 | 0 | 0 |
| *Elymus sibiricus* L. | 0 | 0 | 1 | 0 |
| *Thinopyrum elongatum* (Host) D.R.Dewey | 0 | 0 | 0 | 1 |

**Supplementary Table 2.** The identified specific and shared prescribed herbal species of traditional patent medicine Qingguo Wan based on ITS2, *psbA-trnH*, *matK* and *rbcL*

| Marker | Shared Species | Unique Species | Total |
| --- | --- | --- | --- |
| ITS2 | *Canarium album*  *Lonicera japonica*  *Menispermum dauricum*  *Paeonia lactiflora*  *Panax quinquefolium*  *Platycodon grandiflorus*  *Scrophularia ningpoensis*  *Scutellaria baicalensis* | *Liriope muscari*  *Elymus tsukushiensis*  *Prunus japonica*  *Triticum aestivum*  *Potentilla supina*  *Populus kangdingensis*  *Sanguisorba alpina*  *Ulmus davidiana*  *Rubus pungens*  *Cuscuta australis*  *Pyrus calleryana*  *Artemisia igniaria*  *Pyrus ussuriensis*  *Bupleurum chinense*  *Angelica sinensis*  *Leptopus chinensis*  *Lonicera tragophylla*  *Morus australis*  *Carthamus tinctorius*  *Ephedra intermedia*  *Saccharum sp.*  *Astragalus membranaceus*  *Perilla frutescens*  *Eleutherococcus sessiliflorus*  *Acrocalymma sp.*  *Alternaria alternata*  *Aspergillus penicillioides*  *Aspergillus sp.*  *Boeremia exigua*  *Botryosphaeria dothidea*  *Botrytis cinerea*  *Cladosporium sp.*  *Colletotrichum boninense*  *Coprinopsis cinerea*  *Cystofilobasidium capitatum*  *Cystofilobasidium infirmominiatum*  *Cystofilobasidium macerans*  *Diaporthe sp.*  *Dothideomycetes sp.*  *Fusarium fujikuroi*  *Fusarium oxysporum*  *Fusarium solani*  *Fusarium sp.*  *Leptosphaeria sp.*  *Paraphoma sp.*  *Penicillium polonicum*  *Penicillium salmoniflumine*  *Penicillium sp.*  *Plectosphaerella cucumerina*  *Tausonia pullulans*  *Wallemia mellicola*  *Wickerhamomyces sp.* | 52 |
| *psbA-trnH* |  |  | 0 |
| *matK* |  | *Elymus sibiricus* | 1 |
| *rbcL* |  | *Thinopyrum elongatum* | 1 |

**Supplementary Table 3.** Reads number of prescription components in three commercial samples based on ITS2 sequences

| Latin name | A19 | HSZY146 | HSZY150 |
| --- | --- | --- | --- |
| *Lonicera japonica* | 9087 | 3649 | 9978 |
| *Paeonia lactiflora* | 8152 | 4679 | 4168 |
| *Menispermum dauricum* | 474 | 1835 | 1225 |
| *Scutellaria baicalensis* | 795 | 695 | 919 |
| *Scrophularia ningpoensis* | 7 | 65 | 872 |
| *Platycodon grandiflorus* | 0 | 1126 | 870 |
| *Canarium album* | 344 | 220 | 610 |
| *Ophiopogon japonicus* | 206 | 210 | 84 |
| *Liriope muscari* | 0 | 0 | 41 |
| *Elymus tsukushiensis* | 0 | 71 | 35 |
| *Prunus japonica* | 41 | 0 | 35 |
| *Triticum aestivum* | 0 | 0 | 35 |
| *Potentilla supina* | 0 | 0 | 34 |
| *Populus kangdingensis* | 19 | 13 | 24 |
| *Sanguisorba alpina* | 104 | 0 | 24 |
| *Ulmus davidiana* | 0 | 11 | 22 |
| *Rubus pungens* | 0 | 0 | 22 |
| *Cuscuta australis* | 0 | 0 | 18 |
| *Pyrus calleryana* | 0 | 21 | 16 |
| *Artemisia igniaria* | 6 | 0 | 14 |
| *Pyrus ussuriensis* | 0 | 0 | 14 |
| *Bupleurum chinense* | 0 | 0 | 12 |
| *Angelica sinensis* | 0 | 0 | 10 |
| *Leptopus chinensis* | 0 | 0 | 10 |
| *Lonicera tragophylla* | 0 | 35 | 0 |
| *Morus australis* | 0 | 12 | 0 |
| *Carthamus tinctorius* | 0 | 9 | 0 |
| *Ephedra intermedia* | 0 | 9 | 0 |
| *Saccharum sp.* | 64 | 0 | 0 |
| *Astragalus membranaceus* | 28 | 0 | 0 |
| *Perilla frutescens* | 24 | 0 | 0 |
| *Eleutherococcus sessiliflorus* | 140 | 0 | 0 |

**Supplementary Table 4.** Reads number of prescription components in three commercial samples based on *psbA-trnH* sequences

| Latin name | A19 | HSZY146 | HSZY150 |
| --- | --- | --- | --- |
| *Paeonia lactiflora* | 906 | 463 | 252 |
| *Menispermum dauricum* | 99 | 1000 | 260 |
| *Scutellaria baicalensis* | 55 | 403 | 103 |
| *Platycodon grandiflorus* | 0 | 96 | 33 |
| *Canarium album* | 0 | 196 | 263 |
| *Lonicera japonica* | 444 | 606 | 744 |
| *Scrophularia ningpoensis* | 0 | 32 | 162 |

**Supplementary Table 5.** Reads number of prescription components in three commercial samples based on *matK* sequences

| Latin name | A19 | HSZY146 | HSZY150 |
| --- | --- | --- | --- |
| *Paeonia lactiflora* | 2318 | 1111 | 780 |
| *Menispermum dauricum* | 161 | 1635 | 432 |
| *Scutellaria baicalensis* | 133 | 836 | 418 |
| *Platycodon grandiflorus* | 0 | 192 | 94 |
| *Ophiopogon japonicus* | 0 | 171 | 0 |
| *Canarium album* | 56 | 195 | 191 |
| *Lonicera japonica* | 1156 | 1449 | 1892 |
| *Scrophularia* | 0 | 52 | 256 |
| *Elymus sibiricus* | 0 | 97 | 0 |

**Supplementary Table 6.** Reads number of prescription components in three commercial samples based on *rbcL* sequences

| Latin name | A19 | HSZY146 | HSZY150 |
| --- | --- | --- | --- |
| *Paeonia lactiflora* | 2522 | 1111 | 1029 |
| *Menispermum dauricum* | 157 | 1521 | 602 |
| *Scutellaria baicalensis* | 104 | 466 | 508 |
| *Platycodon grandiflorus* | 0 | 166 | 114 |
| *Ophiopogon japonicus* | 0 | 137 | 0 |
| *Canarium album* | 42 | 127 | 145 |
| *Lonicera japonica* | 1001 | 1021 | 1843 |
| *Scrophularia ningpoensis* | 0 | 41 | 309 |
| *Thinopyrum elongatum* | 0 | 63 | 0 |

**Supplementary Table 7.** Reads number of fungi in five samples based on ITS2 sequences

| Family | Genus | Species | A19 | HSZY146 | HSZY150 | HSZY163 | HSZY175 |
| --- | --- | --- | --- | --- | --- | --- | --- |
| [Morosphaeriaceae](https://www.ncbi.nlm.nih.gov/Taxonomy/Browser/wwwtax.cgi?mode=Undef&id=715496&lvl=3&keep=1&srchmode=1&unlock) | *Acrocalymma* | *Acrocalymma sp.* | 0 | 6 | 0 | 0 | 0 |
| [Pleosporaceae](https://www.ncbi.nlm.nih.gov/Taxonomy/Browser/wwwtax.cgi?mode=Undef&id=28556&lvl=3&keep=1&srchmode=1&unlock) | *[Alternaria](https://www.ncbi.nlm.nih.gov/Taxonomy/Browser/wwwtax.cgi?mode=Undef&id=5598&lvl=3&keep=1&srchmode=1&unlock)* | *Alternaria alternata* | 0 | 45 | 11 | 16 | 0 |
| [Aspergillaceae](https://www.ncbi.nlm.nih.gov/Taxonomy/Browser/wwwtax.cgi?mode=Undef&id=1131492&lvl=3&keep=1&srchmode=1&unlock) | *Aspergillus* | *Aspergillus penicillioides* | 14 | 10 | 16 | 0 | 0 |
| [Aspergillaceae](https://www.ncbi.nlm.nih.gov/Taxonomy/Browser/wwwtax.cgi?mode=Undef&id=1131492&lvl=3&keep=1&srchmode=1&unlock) | *Aspergillus* | *Aspergillus sp.* | 33 | 64 | 76 | 18 | 10 |
| Didymellaceae | *Boeremia* | *Boeremia exigua* | 0 | 30 | 0 | 0 | 0 |
| Botryosphaeriaceae | *Botryosphaeria* | *Botryosphaeria dothidea* | 0 | 0 | 20 | 0 | 0 |
| Sclerotiniaceae | *Botrytis* | *Botrytis cinerea* | 18 | 0 | 20 | 0 | 0 |
| Cladosporiaceae | *Cladosporium* | *Cladosporium sp.* | 0 | 64 | 45 | 34 | 28 |
| Glomerellaceae | *Colletotrichum* | *Colletotrichum boninense* | 0 | 13 | 0 | 16 | 22 |
| Psathyrellaceae | *Coprinopsis* | *Coprinopsis cinerea* | 19 | 0 | 0 | 0 | 0 |
| Cystofilobasidiaceae | *Cystofilobasidium* | *Cystofilobasidium capitatum* | 0 | 8 | 0 | 0 | 0 |
| Cystofilobasidiaceae | *Cystofilobasidium* | *Cystofilobasidium infirmominiatum* | 0 | 16 | 0 | 0 | 0 |
| Cystofilobasidiaceae | *Cystofilobasidium* | *Cystofilobasidium macerans* | 0 | 15 | 0 | 0 | 0 |
| Diaporthaceae | *Diaporthe* | *Diaporthe sp.* | 0 | 0 | 31 | 0 | 0 |
| Other fungi | *Other fungi* | *Dothideomycetes sp.* | 0 | 8 | 0 | 0 | 0 |
| Nectriaceae | *Fusarium* | *Fusarium acuminatum* | 0 | 14 | 10 | 0 | 0 |
| Nectriaceae | *Fusarium* | *Fusarium oxysporum* | 0 | 7 | 0 | 0 | 0 |
| Nectriaceae | *Fusarium* | *Fusarium solani* | 0 | 0 | 30 | 0 | 0 |
| Nectriaceae | *Fusarium* | *Fusarium sp.* | 0 | 0 | 23 | 0 | 0 |
| Leptosphaeriaceae | *Leptosphaeria* | *Leptosphaeria sp.* | 0 | 7 | 0 | 0 | 0 |
| Phaeosphaeriaceae | *Paraphoma* | *Paraphoma sp.* | 0 | 11 | 0 | 0 | 0 |
| Aspergillaceae | *Penicillium* | *Penicillium polonicum* | 30 | 14 | 36 | 6 | 8 |
| Aspergillaceae | *Penicillium* | *Penicillium salmoniflumine* | 0 | 0 | 0 | 4 | 0 |
| Aspergillaceae | *Penicillium* | *Penicillium sp.* | 14 | 35 | 13 | 20 | 15 |
| Plectosphaerellaceae | *Plectosphaerella* | *Plectosphaerella cucumerina* | 0 | 22 | 7 | 0 | 0 |
| Mrakiaceae | *Tausonia* | *Tausonia pullulans* | 0 | 11 | 14 | 0 | 0 |
| Wallemiales | *Wallemia* | *Wallemia mellicola* | 0 | 21 | 10 | 8 | 0 |
| Phaffomycetaceae | *Wickerhamomyces* | *Wickerhamomyces sp.* | 0 | 0 | 0 | 31 | 0 |

**Supplementary Table 8.** DNA extraction quality of Qingguo Wan Sample.

| Dosage form | Drug Name | Sample ID | Concentration（ng/μL） | *A_260/280_* |
| --- | --- | --- | --- | --- |
| Water Honey Pill | Qingguo Pill | A19 | 141.6 | 1.95 |
| Water Honey Pill | Qingguo Pill | HSZY146 | 155.4 | 1.85 |
| Water Honey Pill | Qingguo Pill | HSZY150 | 353.7 | 1.74 |
| Water Honey Pill | Qingguo Pill | HSZY163 | 59.2 | 1.95 |
| Water Honey Pill | Qingguo Pill | HSZY175 | 240.5 | 2.08 |
